# Stricture of the Urethra; Its Complication and Effects

**Published:** 1861-11

**Authors:** 


ART. VI.—*Stricture of the Urethra; its Complication and Effects. A Practical Treatise on the Nature and Treatment of those Affections.* By ROBERT WADE, F.R.S.C., Senior Surgeon to the Westminster General Dispensary; Fellow of the Royal Medical and Chirurgical Society; and late Lecturer on Pathological Anatomy. Fourth edition, with engravings. 8vo., pp. 354. London, 1860.

A WORK which has been honored with a fourth edition, as this has been, requires no recommendation at our hands. Its character is sufficiently established. To prove this, it is only necessary to bestow upon it an attentive perusal, and to read the favorable notices which have been lavished upon it by the British press. Of the numerous treatises on stricture of the urethra that have appeared in Europe, within the last fifteen years, we know of none which, in a practical point of view, is superior to it. If it makes no pretensions to microscopical or historical details, the former of which are now so common in medical writings, it is not, in our judgment, one particle the less valuable. The aim of Mr. Wade has been to produce an eminently practical work, and we congratulate him upon having accomplished, with singular clearness and directness, his object. In his endeavors to do this, he has limited himself chiefly to the results of his own experience, extending through a period of a quarter of a century, in which he has enjoyed ample opportunities, both in public and private practice, of witnessing the effects of the various methods of treating this affection, in all its forms and complications. He has not, of course, altogether neglected the views of other writers; on the contrary, he repeatedly refers to those who have most distinguished themselves in this department of pathology and practice. Great praise is due to Mr. Wade for his laudable endeavors, throughout his treatise, to inculcate the importance of conservative surgery. The following sentence speaks for itself:—

"In this work my great object has been to enforce the adoption of the principles and practice of 'conservative surgery' in the treatment of urethral stricture. Should the views which I have advanced induce others to hesitate before they resort to the more hazardous modes of treatment, which have lately been too prevalent, it is to be hoped that my labors in this branch of the profession may prove useful in saving many patients from the risk incurred by submitting them to operations, not unfrequently fatal, and which, I feel convinced, are seldom required."

We fully concur with the author in these remarks. It may confidently be asserted that in no department of surgery do practitioners evince so much carelessness and recklessness as in this. Experience long ago satisfied us that there are few men in the profession who are really qualified to treat stricture of the urethra upon scientific principles, and frequent observation made since has only served to confirm and strengthen the conviction.

In speaking of the *causes* of this affection, Mr. Wade expresses the belief that there is occasionally a hereditary constitutional tendency to its occurrence, and, in illustration of his opinion, he asserts that he has met with some instances of the kind in his own practice. He refers to one in particular, in which his patient was one of five brothers, who all suffered from urethral obstruction, coming on from the ninth to the fifteenth year. In each case, the disease had to be treated with the bougie. The father had also been a great sufferer from stricture.

Among the exciting causes of this kind of obstruction are, Mr. Wade says, the practice of self-abuse, and the continual discharge of unhealthy urine, containing the oxalate of lime, the lithates or phosphates in excess. In speaking of the habit of self-abuse, he remarks:—

"I have good reason to believe that the pernicious practice of self-abuse is a much more frequent cause of stricture than is generally supposed. In several instances of the kind, in which there had been no sexual intercourse, the strictures which were at the bulb proved more than usually refractory, from the extreme morbid sensitiveness of the entire urethral canal."

He also lays much stress upon the pernicious effects of strong injections in inducing this disease; a fact which is borne out by the experience of every surgeon. The great and pervading causes of the complaint are, of course, gonorrhœa and the remedies employed for its relief.

The author distinguishes strictures into nine varieties, contending that the classification is of great importance in a practical sense. 1. The dilatable stricture. 2. The simple chronic stricture. 3. The impermeable stricture. 4. The irritable stricture. 5. The inflammatory stricture. 6. The elastic stricture. 7. The spasmodic stricture. 8. The traumatic stricture. 9. The cicatricial stricture, from ulceration at the mouth of the urethra. Some of these varieties might possibly be dispensed with; it must certainly be frequently very difficult to distinguish them in practice. We have not time to follow Mr. Wade in his description of them. We cannot, however, forbear to append the following extract:—

"The great majority of cases described as spasmodic stricture, there can be little doubt, result from some degree of inflammation of the mucous membrane at the posterior portion of the urethra; and that it is the irritation from the contact of the urine with the inflamed membrane which excites the surrounding muscles to action, and interferes with their necessary relaxation on the contraction of the detrusor."

Passing by the symptoms and effects of this disease, as topics too well known to require any special notice here, we come to its *treatment*, which is classed under the three heads of dilatation, cauterization, and division.

To the first of these two methods the author expresses himself as highly favorable, believing that, in a large proportion of cases, it is fully adequate to the object, provided it is conducted in a careful and gentle manner, not rudely or forcibly. His opposition to the last proceeding is very strong and decided.

"It is almost needless to remark that the forcible, unscientific manner in which dilatation is occasionally practiced, is the abuse and not the proper use of this most admirable of all methods of treating the generality of urethral strictures. It may be taken for granted that this treatment will be most successful in the hands of those who use all possible gentleness and caution in the introduction of instruments. Let it be taken for granted, also, that it is only by the exercise of great forbearance and a firm resolution to devote the requisite time and attention to every case, which will enable the surgeon to do full justice to this simplest of all methods of procedure."

In regard to the use of instruments for the purpose of effecting dilatation, he holds the following language:—

"If, however, I were restricted to the use of but one kind of instrument, my selection would be the simplest of all, the wax and plaster bougie, varying from a consistence soft enough to receive the impression of a stricture, to such a degree of hardness as would not readily yield to pressure. However inefficient the bougie would undoubtedly prove in some instances, I believe that it would enable me to effect more good, in a large majority of cases, than any other single dilating instrument."

In chapter eighth are discussed the aids to dilatation, and this affords Mr. Wade an opportunity of speaking of his peculiar method of cauterization with caustic potassa, or potassa fusa, a method which he has made peculiarly his own, and which he thinks has hitherto been too much neglected by the profession ; an opinion in which we fully agree with him.

To those who are familiar with the literature of this subject, it is needless to say that the credit of introducing to the notice of the profession the potassa fusa, as a curative agent in this disease, is due to the late Mr. Whately, of London, who described the mode of using it, and the class of cases to which it is more particularly applicable, with great minuteness, in his work entitled "An Improved Method of Treating Strictures in the Urethra." Notwithstanding the able and forcible manner in which he spoke of the remedial powers of this article in the management of this malady, it was but little employed until attention was again called to it by Mr. Wade about thirty years ago. It acts beneficially, as he supposes, first, by relieving irritability and inflammation; secondly, by promoting absorption ; thirdly, by stimulating and diminishing the congested vessels; and, lastly, by dissolving the hardened and contracted strictures. When properly applied, it produces a sensation of heat, with some degree of pain, which, however, is generally very slight and of short duration. The property which it possesses of combining with oily substances and animal mucus, forming a saponaceous compound, modifies its action, and enables it to penetrate the hardened tissues of a stricture, softening them, and thus promoting their absorption in a manner much more effectually than is done by the nitrate of silver. The following is the author's method of using the caustic :—

"Before using the potash, a bougie should be passed down to the stricture, that its distance from the orifice of the urethra may be correctly ascertained. A small piece of the caustic, about the size of a common pin's head to commence with, should be inserted into a hole made in the point of a soft bougie. The caustic should be broken just before it is required, and the inner or dark part selected, as the outer portion is usually less efficient, as it is commonly converted into a whitish crust of carbonate of potash. Two notches should be made in the armed bougie, as directed by Mr. Whately—one marking the exact distance of the stricture; the other, an inch beyond; so that its progress, as it enters the obstruction, may be accurately observed. The bougie should be moulded with the finger round the potassa fusa, so that it may be securely fixed; but to insure the action of the caustic, instead of being below the level of the hole of the instrument, as recommended by Mr. Whately, its points should be fairly exposed to enable it to act upon the stricture.

"The armed bougie should, of course, be well oiled before its introduction; and if the points of the caustic are well covered with lard, there need be no fear of its acting before it reaches the stricture. The bougie should be gently pressed against the stricture for a minute or two if impermeable, and then withdrawn. When the caustic is applied to permeable obstructions, the bougie should be passed three or four times over the whole surface of the stricture. To impermeable strictures the caustic should be applied with greater caution than to such as are permeable; for should retention of urine occur, it will be more easily relieved in the latter than in the former. It usually happens that, after one or two applications of the caustic, the bougie will be found to enter the obstruction."

The best time for the application is just before going to bed; the bladder should be previously emptied, and an anodyne enema may be administered if there be danger of rigors or retention of urine. The free use of the caustic is generally followed by more or less of a slimy, muco-purulent discharge, at first usually slightly tinged with blood, but soon assuming a turbid, whitish hue. The frequency with which the application is to be repeated depends upon its effect and the nature of the case. In old chronic strictures, Mr. Wade has often used it advantageously every second or third day, and in some instances, under peculiar circumstances, even at shorter intervals.

The cases in which he has found the remedy more particularly applicable are, first, strictures of a cartilaginous hardness, impervious to instruments; secondly, strictures of long standing, which, although admitting the passage of a small bougie, bleed more or less freely on its introduction; and, lastly, irritable strictures, and such as have a marked tendency to spasm.

He positively asserts that the use of the remedy has never, in his hands, produced a stricture, or aggravated any while under treatment; and he adds that he is thoroughly convinced that it creates less tendency to re-contraction in the more aggravated forms of the complaint than when it is treated simply by dilatation. In a word, his confidence in the use of the caustic is almost unbounded.

We have not space to follow the author through his remarks upon the other methods of treatment, especially external division of strictures; nor can we accompany him in what he says in regard to the complication of these affections. We have been particularly desirous of presenting to the reader a full view of his own method, and, having done this, we are reluctantly compelled to bring our notice to a close. We cannot do so, however, without tendering to Mr. Wade our cordial acknowledgments for the pleasure and instruction we have derived from the perusal of his work, and expressing the conviction that it is one of the most able, practical treatises on the subject in any language. One of the most valuable features of it is the narration of numerous cases of this disease, illustrative of its nature and symptoms, and of the author's peculiar mode of treatment. They are drawn up with great care, and bear ample testimony to the immense opportunities which he has enjoyed for studying this complaint in all its forms and complications. We trust that the work will be duly appreciated on this side of the Atlantic, and that our hospital surgeons will give a fair trial to a method of treatment of the favorable effects of which we can speak from personal observation. The mechanical execution of the book is excellent.

